# Compression rates of microbial genomes are associated with genome size and base composition

**DOI:** 10.1186/s44342-024-00018-z

**Published:** 2024-10-10

**Authors:** Jon Bohlin, John H.-O. Pettersson

**Affiliations:** 1 Norwegian Institute of Public Health, Domain for Infection Control, Section for Modeling and Bioinformatics, Oslo, Norway; 2 Zoonosis Science Center, Clinical Microbiology, Department of Medical Sciences, University of Uppsala, 751 85 Uppsala, Sweden; 3 Clinical Microbiology and Hospital Hygiene, Uppsala University Hospital, 751 85 Uppsala, Sweden; 4 Department of Microbiology and Immunology, Peter Doherty Institute for Infection and Immunity, University of Melbourne, Melbourne, VIC Australia

**Keywords:** MBGC, ZPAQ, Microbial Genomics, Compression, Information potential, Base composition

## Abstract

**Background:**

To what degree a string of symbols can be compressed reveals important details about its complexity. For instance, strings that are not compressible are random and carry a low information potential while the opposite is true for highly compressible strings. We explore to what extent microbial genomes are amenable to compression as they vary considerably both with respect to size and base composition. For instance, microbial genome sizes vary from less than 100,000 base pairs in symbionts to more than 10 million in soil-dwellers. Genomic base composition, often summarized as genomic AT or GC content due to the similar frequencies of adenine and thymine on one hand and cytosine and guanine on the other, also vary substantially; the most extreme microbes can have genomes with AT content below 25% or above 85% AT. Base composition determines the frequency of DNA words, consisting of multiple nucleotides or oligonucleotides, and may therefore also influence compressibility. Using 4,713 RefSeq genomes, we examined the association between compressibility, using both a DNA based- (MBGC) and a general purpose (ZPAQ) compression algorithm, and genome size, AT content as well as genomic oligonucleotide usage variance (OUV) using generalized additive models.

**Results:**

We find that genome size (*p* < *0.001*) and OUV (*p* < *0.001*) are both strongly associated with genome redundancy for both type of file compressors. The DNA-based MBGC compressor managed to improve compression with approximately 3% on average with respect to ZPAQ. Moreover, MBGC detected a significant (*p* < *0.001*) compression ratio difference between AT poor and AT rich genomes which was not detected with ZPAQ.

**Conclusion:**

As lack of compressibility is equivalent to randomness, our findings suggest that smaller and AT rich genomes may have accumulated more random mutations on average than larger and AT poor genomes which, in turn, were significantly more redundant. Moreover, we find that OUV is a strong proxy for genome compressibility in microbial genomes. The ZPAQ compressor was found to agree with the MBGC compressor, albeit with a poorer performance, except for the compressibility of AT-rich and AT-poor/GC-rich genomes.

**Supplementary Information:**

The online version contains supplementary material available at 10.1186/s44342-024-00018-z.

## Background

Microbial genomes vary substantially with regard to genome size and base composition [1]. Due to Chargaff’s parity rules [2], base composition in microbes can be summarized as the percentage % of (A)denine% + (T)hymine% (%AT), due to their similar frequencies. Alternatively, it can be summarized as 100%—(%AT) = (G)uanine% + (C)ytosine% (%GC). In general, genomic %AT (AT) is negatively associated with genome size [3, 4]. Within microbial species, however, genomic AT has been found to increase with genome size [5]. While free-living and soil dwelling microbes tend to have larger genomes with less AT (GC rich) [6], intracellular symbionts [7], and to a lesser extent pathogens [8], have smaller, AT rich genomes due to reductive evolution [9]. A negative association has also been found between genomic AT content and oligonucleotide frequencies, sometimes referred to as oligonucleotide usage variance (OUV) [10–12], i.e., genome-wide frequencies of “DNA words” consisting of a fixed number of nucleotides (usually 3 (codons) [13] or 4 (tetranucleotides)) [11]. This means that the frequency of genomic DNA words is more associated with base composition the more AT rich the bacterial genomes are [14]. Here, OUV is the averaged sum of the squared differences between all possible genomic tetranucleotide frequencies subtracted by their corresponding expected tetranucleotide frequencies. As such, OUV literarily describes the variance of genomic tetranucleotide frequencies. The expected tetranucleotide frequencies are estimated by the respective tetranucleotide word’s individual genomic nucleotide frequencies. That is, if *f(AGCT)* represents the genomic frequencies of the tetranucleotide word “AGCT,” then the estimated expected frequency of the word “AGCT” will be *f(A)f(G)f(C)f(T)*. OUV thus represents the average squared difference, or variance, taken over the frequencies of all 4^4^ = 256 possible combinations of tetranucleotide words subtracted by the expected occurrence of the corresponding tetranucleotide, e.g., (*f(AGCT) – f(A)f(G)f(C)f(T)*)^2^ [12]. Genomes with biased tetranucleotide frequencies, for one reason or another, will have higher OUV as some DNA words will differ greatly from what is expected from the base composition (i.e., AT/GC content) alone. High OUV will thus likely represent genomes having been subjected to strong selective pressures, either positive or negative [15]. Organisms with low OUV, i.e., oligonucleotides that are more predictable from genomic base composition, have been subjected to more relaxed selective pressures [15]. It has been shown that AT rich genomes tend to consist of oligonucleotides that have more similar frequencies to that of the product of the corresponding single nucleotide frequencies and thus tend to have lower OUV than AT poor microbes [11]. AT poor (sometimes referred to as GC rich) microbes, on the other hand, often have more selective tetranucleotide word usage than AT rich ones resulting in higher OUV [15]. These discrepancies between AT rich and AT poor genomes, with respect to genome-wide tetranucleotide word frequencies, have been linked to selective pressures [11]; AT rich genomes appear historically to have been subjected less to selective pressures than AT poor genomes [16, 17]. For microbial symbionts, which are often AT rich, this might be, to some extent, explained by easier access to metabolites from the host [18] and/or lower effective population size [19]. It is also anticipated that genomes being subjected to relaxed selective pressures, such as symbionts, may accumulate genome-wide mutations to a larger degree than organisms subjected to strong selective pressures [7]. Accumulation of genome-wide random mutations is therefore assumed to result in tetranucleotide word frequencies more in line with AT/GC content [12].

As mentioned above, AT rich genomes have often been found to be more associated with symbiotic and pathogenic microbes with the latter having slightly larger genomes [8]. AT poor genomes are often found in environments outside of the host [13]. Since genomic AT content correlates negatively with nitrogen abundance [20], many microbial species with AT poor genomes are often found in soil [13]. Hence, it is assumed that OUV differences are reflective of the selective pressures mediated by the respective environments [12]. Non-host environments are likely challenging requiring a diverse set of genes for utilizing different types of nutrients for survival. Stronger selective pressures, in particular purifying selection and/or frequent bottleneck events, may therefore favor species that are more competitive with the ability to survive in diverse and dynamic environments. Under such circumstances, there could be a strong selection for more specific DNA patterns resulting in more biased genome-wide tetranucleotide word frequencies, reflected by higher OUV [21].

The extent to which a genome can be compressed may reveal important characteristics about its complexity [22]. For instance, genomes that can be compressed carry a larger information potential than those that cannot, which, in turn, are more random with a lower information potential [23]. Genome-wide oligonucleotides are more easily compressed if they exhibit specific patterns or some degree of systematic bias. Bias resulting from selection for specific genome-wide DNA patterns is reflected by higher OUV [21]. The genomes of such organisms could thus be easier to compress than organisms with low OUV that do not show any genome-wide DNA patterns. In this sense, AT poor/GC rich genomes, with higher OUV, are expected to carry a higher information potential than more AT-rich genomes.

The most common general-purpose compression algorithm (often referred to as LZ after the inventors Abraham Lempel and Jacob Ziv [24]) attempts to reduce a string of data by searching for repeating patterns or sub-strings and extending these as much as possible. Repeating substrings or patterns are then indexed, i.e., replaced by smaller symbols, thereby reducing string size. Finally, a dictionary containing the symbols representing specific sub-strings is added to the compressed file for use in decompression. If a string contains many different patterns that require symbols of varying size for representation, the Huffman algorithm will make sure that the most frequent patterns, or sub-strings, are assigned progressively shorter representations [25]. The size of such representations therefore correlates negatively with occurrence frequency. Hence, strings consisting of many repetitive patterns are easier to compress than strings containing fewer such patterns [24]. Most modern general-purpose compressors consist of several algorithms and steps to reduce the size of a string of data [26].

Recently, compression algorithms were introduced that take DNA specific properties into account [26]. In particular, the Multiple Bacterial Genome Compressor (MBGC) considers DNA patterns and their reverse complements [27]. As such, MBGC achieves greater compression ratios for DNA-based strings than other non-DNA based compressors.

In theoretical computer science, an equivalence relation has been established between compressibility and a definition of randomness [28]; the more random and unpredictable a string of letters (such as A, G, C and T) is, the harder it is to compress. A string consisting of a few substring patterns is therefore more difficult to compress. As such, by exploring the redundancy of microbial genomes using AT content and OUV, a measure of genomic randomness is also attained.

Since AT content in microbial genomes is, in one way or another, associated with genome size and OUV, the purpose of this project was to examine whether this association could also be extended to genome sequence complexity as measured by compression rate. Hence, we wanted to explore if there is an association between AT, genome size and OUV on one hand and the compressibility of the corresponding microbial genome on the other. To test this hypothesis, 4713 microbial genomes, representing 1508 bacterial genera, were downloaded from the NCBI RefSeq database to minimize species- and strain-specific bias. These genomes were compressed using two different algorithms: ZPAQ, which is widely regarded as the best general purpose compression algorithm [26], and MBGC, a recent compression algorithm specialized for microbial genomes [27]. The compression rates obtained from both these algorithms for the 4713 RefSeq genomes were subsequently regressed on corresponding genomic AT content, OUV, and genome size using a generalized additive model [29]. The models were also adjusted for taxonomic relatedness.

## Results

**Fig. 1:**
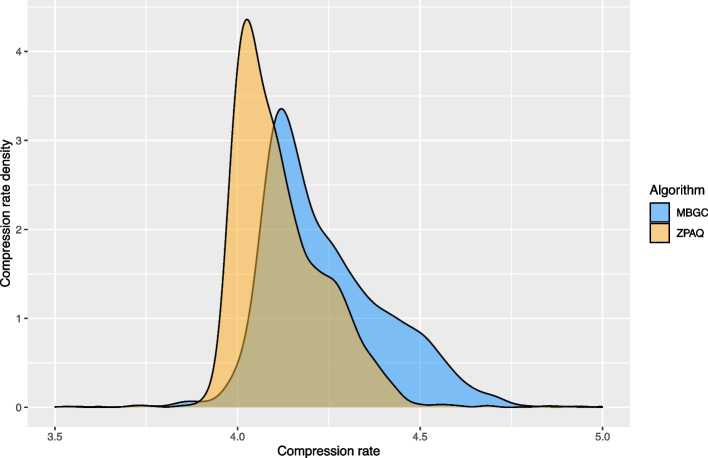
Genomic compression rate. A distribution plot based on the genomic compression rate (genome size/compressed genome size, horizontal axis) for 4713 RefSeq genomes based on both ZPAQ and MBGC compression algorithms

A total of 4713 microbial genomes were compressed using both ZPAQ and MBGC algorithms [26, 27]. Compression rate was calculated as genome size divided by compressed genome size (see Fig. 1).

**Fig. 2:**
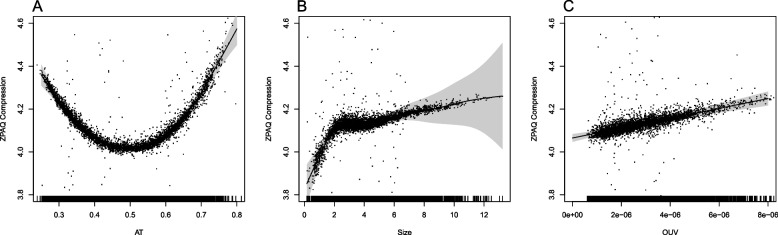
ZPAQ compression rate regressed on genomic AT content, genome size, and OUV. The figure shows the predictors (horizontal axis): AT content (edf = 6.3, *p* < *0.001*) (**A**), genome size (edf = 7.1, *p* < *0.001*) (**B**), and OUV (edf = 1, *p* < *0.001*) (**C**), from a GAMM regression model with ZPAQ-based genomic compression rate as the outcome (vertical axis). Effective degrees of freedom (edf) indicate degree of smooth non-linearity for edf > 1. The model also included a random slope effect of genome size with respect to phylogeny (genus)

A generalized additive mixed-effects regression model (GAMM) was fitted with ZPAQ compression ratio as the outcome and genomic AT content, genome (chromosome) size, and OUV as predictors represented by splines, to compensate for putative non-linear trends, for all 4713 genomes (see Fig. 2). In addition, taxonomic genus was added as a random effect with respect to genome size, which was established by examining differences with respect to the Akaike Information Criterion (AIC) goodness-of-fit statistic between tested models (see Table 1 and Section 4 for details) [30, 31].

**Table 1:** AIC goodness-of-fit statistic for GAMM models

Model	ZPAQ	MBGC	MBGC-ZPAQ
*Full*	− 3082	− 2997	− 21,157
*No random effects*	− 2053	− 1993	− 20,868
*Null*	− 725	445	445

The GAMM model indicated that there was a positive association between ZPAQ compression ratio and the three smooths representing respectively genomic AT content (*p* < *0.001*), genome size (*p* < *0.001*), and OUV (*p* < *0.001*). Both genomic AT content and genome size exhibited substantial non-linear trends, as measured using effective degrees of freedom (edf, edf > 1 indicates non-linearity), with respective edf = 6.3 and edf = 7.1. OUV, on the other hand, did not exhibit any non-linear trends (edf = 1) and was found to be positively associated with the ZPAQ compression rate. The AIC for this model (see also Table 1) was − 3082 as compared to AIC = − 2053 for the model with the same predictors but without the random slope effects (i.e., lower AIC is better). The AIC for the null model (the outcome regressed on a constant) was − 725.

**Fig. 3:**
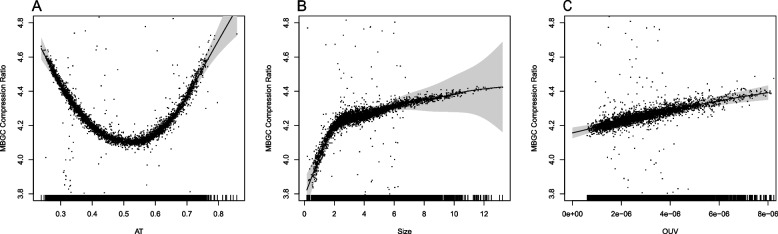
MBGC compression rate regressed on genomic AT content, genome size, and OUV. The figure shows the predictors (horizontal axis): AT content (edf = 7.1, *p* < *0.001*) (**A**), genome size (edf = 7.5, *p* < *0.001*) (**B**), and OUV (edf = 2.0, *p* < *0.001*) (**C**), from a GAMM regression model with MBGC-based genomic compression rate as the outcome (vertical axis). Effective degrees of freedom (edf) indicate degree of smooth non-linearity for edf > 1. The model also included a random slope effect of genome size with respect to phylogeny (genus)

An additional GAMM model was fitted with the same predictors and random effects as for the ZPAQ compression model above but with the MBGC compression rate as the outcome instead (see Fig. 3). Again, both AT content and genome size were found significant (*p* < *0.001*) with considerable non-linear splines: edf = 7.1 and edf = 7.5, respectively. For this model, non-linear trends were also observed for the smooth representing the OUV predictor (edf = 2.0). Once more, the model obtained a better AIC (− 2997, see Table 1) when a random slope of genome size with respect to genus was included in addition to the fixed effect predictors AT, genome size, and OUV. For the same model but without the random slope, AIC = − 1993 and AIC = 445 for the null model (only outcome). It can be seen from both Figs. 2 and 3 that while there is a clear positive association with respect to genome size and OUV on one hand and for both ZPAQ and MBGC genome compression ratios on the other, the trend with regards to AT content is less clear due to the parabolic trend.

**Fig. 4:**
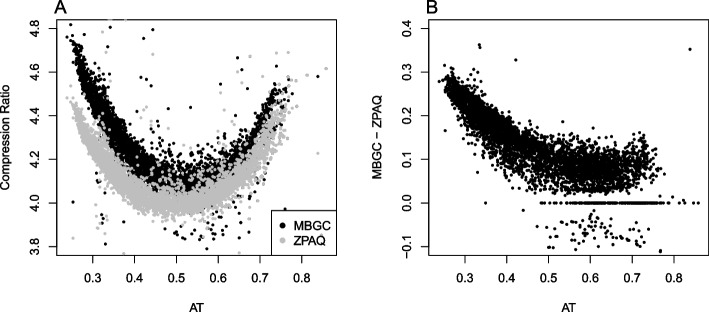
MBGC and ZPAQ compression rate differences with regard to AT content. **A** Genomic compression ratios (vertical axis) for both MBGC and ZPAQ algorithms plotted against genomic AT content (horizontal axis). **B** The compression rate difference between the two algorithms (vertical axis) with regard to genomic AT content (horizontal axis)

**Fig. 5:**
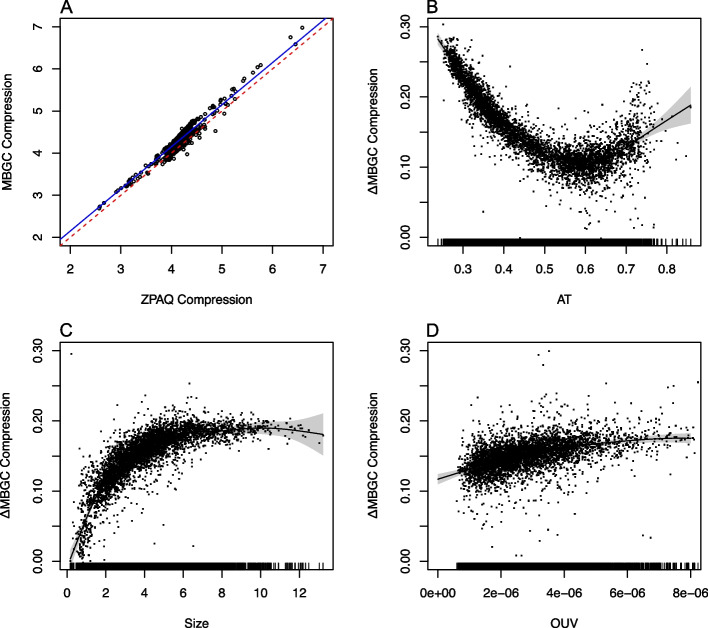
Regression model of compression rate differences. The figure demonstrates remaining effects of the MBGC compression rate (vertical axis) model after the effects from the ZPAQ compression rate model (**A**, horizontal axis) have been adjusted away with regression line colored in blue (red dashed line has intercept 0 and slope 1). **B** The remaining compression rate difference (vertical axis) on genomic AT content (horizontal axis, edf = 7.7, *p* < *0.001*), **C** genome size (horizontal axis, edf = 8.3, *p* < *0.001*), and **D** OUV (horizontal axis, edf = 4.9, *p* < *0.001*). Genome size with respect to phylogeny (genus) was additionally included as a random slope. Effective degrees of freedom (edf) indicate degree of non-linearity (i.e., edf > 1) in smoothing spline

Considering the results obtained above, we wanted to explore the relationship between the two compression algorithms. Not only does Fig. 4 indicate that MBGC is, on average, better at compressing genomes than the ZPAQ algorithm but also that AT poor/GC rich genomes are considerably better compressed with the MBGC method. To assess the difference between the compression algorithms more formally, the GAMM model with MBGC as the outcome was refitted but now with the ZPAQ compression rate as an additional linear covariate, leaving the rest of the model, as described above, unchanged. The model describes the compression rate differences between the algorithms with regard to AT content, genome size, and OUV (see Fig. 5B–D). AIC improved considerably with – 21,157 for this model (AIC = − 20,878 without random effects; see also Table 1). Figure 5 shows an approximate linear relationship between the compression ratios of MBGC and ZPAQ (*p* < *0.001*) and that there is a, largely negative, association between the MBGC compression rate and AT content (*p* < *0.001*), suggesting that compression of AT poor genomes is visibly improved with the MBGC algorithm as compared to the ZPAQ algorithm. Slight improvements over the ZPAQ algorithm can also be observed for genome size and OUV. Excepting the ZPAQ compression ratio, which was assumed to be linear, AT content, genome size, and OUV were all non-linear terms with edf equal to 7.7, 8.3, and 4.9, respectively.

It can also be noticed in Fig. 5 that the red dashed line, which represents a regression line with slope 1 and intercept 0, was slightly lower than the (blue) regression line, which represents the model intercept (*p* < *0.001*) for the MBGC compression ratio regressed on the ZPAQ compression rate (and adjusted for the other predictors and the random slope effect mentioned above). The model intercept indicates that the MBGC compressor achieves, on average, a 0.15 compression ratio improvement (approximately 3%) over the ZPAQ compressor for microbial genomes.

## Discussion

OUV is a measure of genomic oligonucleotide frequency discrepancy from what is expected based only on the corresponding genomic nucleotide frequencies (i.e., genomic AT content). Surprisingly, OUV exhibited a strong correlation with both ZPAQ and MBGC compression ratios (see respective Figs. 2 and 3). Higher values of OUV indicate that oligonucleotide frequencies deviate from expected based on the corresponding individual nucleotide frequencies. Low OUV, on the other hand, suggests that oligonucleotide frequencies are more predictive from the corresponding individual nucleotide frequencies. Low OUV may thus be indicative of microbial genomes whose oligonucleotides have historically been subjected to relaxed selective pressures as compared to those with high OUV. Alternatively, low OUV may also, at least to some extent, be reflective of microbes with genomes containing a higher number of accumulated, presumably random, mutations.

Systematic bias in oligonucleotide usage may facilitate compression, particularly for the oligonucleotides that occur more frequently than expected. From the results presented here for the MBGC compression rates, this is more prevalent in AT poor genomes as seen in Figs. 4 and 5. Indeed, a negative association has previously been found between OUV and AT content both within [14] and between genomes [11]. In general, AT poor genomes appear to have more homogeneous oligonucleotide usage [32]. As mentioned previously, biased genome-wide oligonucleotide usage facilitates compression, something that is reflected in the results presented here. The fact that MBGC compressed AT poor genomes better than ZPAQ indicates that oligonucleotides occur with similar frequency to their reverse complements increasingly more than for AT rich genomes. Hence, biased occurrence of genomic oligonucleotides is increasingly more similar to the occurrence of the corresponding reverse complemented oligonucleotides in genomes with decreasing genomic AT.

As mentioned above, since OUV is lower in AT rich genomes, oligonucleotide frequencies are more correlated to the frequencies of oligonucleotides composed of the corresponding individual nucleotides. In other words, genomic nucleotide frequencies tend to be more predictive of the genomic oligonucleotide frequencies. This could potentially indicate that species with AT rich genomes have historically accumulated more mutations than species with AT poor/GC rich genomes. Weaker purifying selection may thus have resulted in accumulation of mutations in AT rich genomes to a much larger extent than in AT poor genomes.

The performance of the ZPAQ algorithm was on average inferior to the MBGC algorithm, and no compression rate differences could be detected between AT rich and AT poor genomes. Both ZPAQ and MBGC algorithms are similar [27], except for the fact that MBGC takes into considerations oligonucleotides and their reverse complements. The difference in compression rate observed with MBGC between AT rich and AT poor genomes must therefore point to trend differences in oligonucleotide usage. More specifically, AT poor genomes have more homogeneous frequencies of abundant oligonucleotides and their reverse complements than AT rich genomes. For both compression algorithms, the lowest compression rates were nevertheless obtained for the genomes with similar AT/GC content (i.e., %AT/%GC approaching 50%), likely because more oligonucleotide frequencies are less predictable due to the similar nucleotide individual frequencies.

AT rich microbial genomes tend to have smaller genomes than GC rich genomes [3]. The reason is not completely understood, but as was seen in Figs. 2 and 3, the smaller genomes are also harder to compress, at least those with genome sizes below 2 million base-pairs (mb). Symbionts and pathogens often have smaller genomes due to genome reduction [7]. Reduction in genome size is often preceded by an accumulation of mutations, which go hand in hand with increased AT content and number of pseudogenes [7]. The reason that mutation accumulation is often positively associated with AT content is that most mutations are in the direction from cytosine to thymine [16]. Microbes with AT poor genomes, however, are often found outside hosts and have larger genomes with a more diverse set of genes [33]. An important difference between adenine and thymine base-pairs on one hand and guanine and cytosine base-pairs on the other is that the latter pair requires three hydrogen bonds instead of two for the former. Hence, G-C base-pairs are stronger than A-T but require more stacking energy [19, 34]. This suggests that genomic AT content is, at least to some extent, also a historical record of selective pressures, or lack thereof, having acted on a species’ genome [33, 34].

Formal definitions of the concept of randomness are relatively recent [28]. It has been shown that randomness is equivalent with compression [35]; the more random a string of characters is, the harder it is to compress and vice versa. Random sequences are also connected to the notion of information. The more random a string of characters is the less information potential is carried by it (i.e., entropy increases) [23]. It is shown here that microbes with smaller genomes (i.e., typically less than 2 mb) are harder to compress than microbes with larger genomes. Moreover, AT poor genomes are easier to compress than AT rich, at least with the MBGC compression algorithm. Finally, OUV, which is a measure strongly connected to information and entropy [21], is positively associated with both ZPAQ and MBGC compression rates. As such, smaller AT-rich genomes with lower OUV, often found to be host associated, tend to contain less information potential, at least with respect to Shannon entropy [21, 23], than larger AT poor genomes with higher OUV typically found in the environment.

## Methods

In total 4829 microbial genomes were downloaded from NCBI’s RefSeq database [36] (https://www.ncbi.nlm.nih.gov/refseq/) on February 23, 2024. To obtain a prokaryotic population consisting of representative samples as much as possible, only RefSeq genomes were considered. The genomic data consisted mostly of one genome from each species, excepting only species with substantial genomic differences, within a genus. There were 114 samples of *Wolbachia endosymbiont*, of which 113 were removed to reduce bias from a single species. Three additional genomes were removed due to an unusual high number of repeats resulting in over 30 times compression ratios for both ZPAQ and MBGC compressors. The exceedingly high compression ratios negatively impacted model assumptions and were therefore removed. These included an unnamed endosymbiont of the deep-sea mussel *Bathymodiolus septemdierum* (accession: AP013042.1), *Enterobacter lignolyticus* (CP012871.1), and *E. ludwigii* (CP017279.1). In total, 4,713 genomes were available for analysis. All plasmids were removed from all species/genomes so that only chromosomes were used for the downstream analyses. Genomes consisting of multiple chromosomes were concatenated into one file.

The genomes were compressed into ZPAQ format using “lrzip” v. 0.651 with the –zpaq option [26]. For the MBGC compressor, “mbgc” v. 1.2.1 [27] was used (https://github.com/kowallus/mbgc). Compression ratio was calculated as genome (i.e., total chromosome) size/compressed file size for each genome and for both compressors.

The AT content, OUV and genome size of these genomes were computed from in-house scripts; AT content as the number of A + T nucleotides/total number of A + T + G + C nucleotides. Genome size was calculated as the total number of nucleotides. OUV was calculated as the average squared difference between genomic tetranucleotide frequencies and the product of its individual nucleotide frequencies. That is:

$$OUV=\frac1{N-1}\sum_{XYZW}\left(f\left(XYZW\right)-f\left(X\right)f\left(Y\right)f\left(Z\right)f\left(W\right)\right)^2$$

where *XYZW* represents nucleotides for every possible genomic tetranucleotide from the alphabet *A*, *G*, *C* and *T* (e.g., *f(ATGC) and f(A)f(T)f(G)f(C)*). *N* designates the number of possible oligonucleotide combination (i.e., *N* = *4*^*4*^ = *256* for tetranucleotides).

To explore whether genomic compression ratio, from either ZPAQ or MBGC algorithms, was associated with genome size, AT content, and OUV, we employed generalized additive mixed effects models (GAMM) [29] to account for putative non-linear associations between outcome and predictors and non-constant variance differences between phylogenetic groups. It has previously been demonstrated that adding a taxonomic group below genus level has negligible impact on the models focusing on genomic base composition in microbes [15, 37]. Genus was therefore added as a random intercept effect since there was only one genome for each species. A random slope effect with respect to genome size resulted in the best fitted model. All models were first estimated using Maximum Likelihood (ML) so that it would be possible to assess and compare goodness-of-fit with the Akaike Information Criterion (AIC) [31]. The lowest AIC indicates the best model. The best models for both regression models with compression ratio (ZPAQ/MBGC) as outcome and genomic AT content, OUV, and genome size as outcome, together with a random slope of genome size with respect to genus, obtained AIC = − 3082 (ZPAQ)/ − 2997 (MBGC). Without random effects but the same predictors the models obtained, AIC = − 2053 (ZPAQ)/ − 1993 (MBGC). For the null models, − 725 (ZPAQ)/445 (MBGC). To compare the difference between the MBGC compression ratio and the ZPAQ compression ratio with respect to the abovementioned predictors, the ZPAQ compression rate was added to the MBGC compression rate model described above as a linear predictor. Hence, Fig. 5 shows the remaining effects of MBGC compression rate model after adjusting away the effects from the ZPAQ compression rate. All final mixed-effect type models were estimated using restricted maximum likelihood (REML); therefore, all results presented (except for AIC) are from these models [30]. All statistical analyses were performed in R v. 4.3.1 [38], GAM regression was carried out with the “mgcv” library [29], while the mixed effect GAM (GAMM) was performed with the “GAMM4” library that estimates random effects using the “lme4” library [39]. All figures were also made with R.

## Conclusion

We demonstrate here that microbes with smaller genomes tend to be harder to compress than microbes with genome sizes approximately above 2 mb. Moreover, we found a clear and surprising positive association between OUV and compression rate suggesting that increasing oligonucleotide usage variance is a proxy for genome redundancy in microbes. Since the OUV measure is related to Shannon entropy, there is a positive association between genome redundancy and information potential.

The MBGC method compressed microbial genomes to a higher rate than the ZPAQ algorithm, on average. Moreover, MBGC compressed AT poor genomes significantly better than the ZPAQ compressor. The MBGC algorithm’s ability to obtain a progressively higher compression rate for genomes with decreasing AT content is likely because the method considered both oligonucleotides and their reverse complements. These findings suggest that organisms with AT poor/GC rich genomes have higher than expected occurrences of particular oligonucleotides and their respective reverse complements than AT rich genomes. Since compression is tightly linked to randomness, smaller, AT rich genomes with low OUV, often found to be host-associated, appear to have accumulated more random mutations, on average, and thus exhibit lower information potential, than microbes with larger, AT poor/GC rich genomes and higher OUV frequently found in soil and the environment.

## Supplementary Information

Additional file 1. An Excel file containing all data needed to reproduce the findings reported.

## Data Availability

All genomes were downloaded from the NCBI Reference Sequence Database (RefSeq, https://www.ncbi.nlm.nih.gov/refseq/). All data needed to reproduce the findings reported have been included as an Excel spreadsheet in Additional file 1.

## Data availbility

All data used for analysis have been included in an Excel file as supplementary information.
